# Pricing the urban cooling benefits of solar panel deployment in Sydney, Australia

**DOI:** 10.1038/srep43938

**Published:** 2017-03-06

**Authors:** S. Ma, M. Goldstein, A. J. Pitman, N. Haghdadi, I. MacGill

**Affiliations:** 1 ARC Centre of Excellence for Climate System Science and Climate Change Research Centre, University of New South Wales, Sydney, Australia; 2 Babson College, MA, USA; 3 School of PV and Renewable Energy Engineering and Centre for Energy and Environmental Markets, University of New South Wales, Sydney Australia; 4 Centre for Energy and Environmental Markets and School of Electrical Engineering and Telecommunications, University of New South Wales, Sydney, Australia

## Abstract

Cities import energy, which in combination with their typically high solar absorption and low moisture availability generates the urban heat island effect (UHI). The UHI, combined with human-induced warming, makes our densely populated cities particularly vulnerable to climate change. We examine the utility of solar photovoltaic (PV) system deployment on urban rooftops to reduce the UHI, and we price one potential value of this impact. The installation of PV systems over Sydney, Australia reduces summer maximum temperatures by up to 1 °C because the need to import energy is offset by local generation. This offset has a direct environmental benefit, cooling local maximum temperatures, but also a direct economic value in the energy generated. The indirect benefit associated with the temperature changes is between net AUD$230,000 and $3,380,000 depending on the intensity of PV systems deployment. Therefore, even very large PV installations will not offset global warming, but could generate enough energy to negate the need to import energy, and thereby reduce air temperatures. The energy produced, and the benefits of cooling beyond local PV installation sites, would reduce the vulnerability of urban populations and infrastructure to temperature extremes.

Cities account for about 2% of land area, consume 60–85% of the world’s energy^1^, and are responsible for about 70% of the world’s CO_2_ emissions^2^. Countries are becoming increasingly urbanized and the global population is projected to be 70% urban by 2050^1^. Responding to these trends, the 2015 United Nations Climate Change Conference in Paris (COP21) highlighted the importance of cities to climate action^3^. Anthropogenic emissions of greenhouse gases warm our cities, but the character of the urban form drives a surface energy balance that is characterized by a higher fraction of available energy exchanged as sensible heat^4^, which adds to localized warming. In addition, substantial energy, in the form of electricity, gas, solid fuels and oil, from remote locations is directly imported to supply residential, commercial and industrial energy services^5^,^6^, which ultimately generates heat that adds further to localized warming.

There are numerous strategies to reduce the vulnerability of cities and their populations to heat^7^ including surface geo-engineering by painting surfaces white to reflect more incoming solar radiation^8^,^9^,^10^ and vegetating roofs^11^,^12^,^13^. Costs associated with these methods can be high, and they require on-going maintenance, but the costs can be offset by the environmental benefits. One geo-engineering option that has potential as part of a strategy of cooling cities is the large-scale deployment of solar photovoltaic (PV) systems^14^,^15^. It is common to explore the potential for solar power systems (primarily PV although concentrating solar plant are also an option at utility scale) to generate electricity, with associated direct economic benefits, and indirect benefits on CO_2_ emissions and climate^16^. For example, large-scale installation of solar panel over desert regions has been shown to cool regional climate and have relatively benign impacts on global climate^16^. The potential is enormous, with estimates of harvestable solar energy worldwide in the range^17^ ~400 to 8.800 TW (1 TW = 10^12^ J s^−1^).

PV panels are specifically designed to have low albedo, hence large-scale urban installations has the potential to increase the absorption of solar radiation. This might add to the solar energy loading of a city, increasing the UHI effect. However, if a city can generate enough energy to meet its local demands via solar energy, including any additional energy demands consequential on the lower albedo associated with the solar panels, then no energy needs to be generated remotely and then imported. This avoidance of energy importation reduces the total energy added to the system because the energy absorbed by the solar panel does not directly warm an urban surface. Instead, it is taken and used to generate electricity that is then ultimately returned to the environment as heat over a longer period of time and which replaces energy that otherwise needs to be imported. Solar panels can therefore cool daytime temperatures in a way similar to increasing albedo via white roofs, but unlike these other methods solar panels also produce valuable electricity. Ultimately, the reduction in, or even elimination of, imported energy reduces the city’s total energy footprint, provides a financial return to the PV system owners and might cool a city to provide a free environmental benefit and even financial saving to those in the city without solar panels. The potential of this cooling to reduce risks to infrastructure and human health could also be considerable. We examine the scale of these benefits and estimate separately the value of the temperature reduction and the electricity creation.

## Impacts of solar panels on winter and summer temperatures

We use the Weather Research Forecast (WRF 3.7.1) model to simulate the January (summer) and July (winter) climate of Sydney in 2007 and 2009 with and without the implementation of various solar panel configurations (see Methods and Supplementary Figure S1). The model is initialized and updated at the lateral boundary using ERA-Interim reanalysis at 6-hourly intervals^18^. In our experiments, the percentage of the roof area depends on the urban land use intensity (Supplementary Figure S1) and we assume roofs are fully covered by solar panels. Clearly, complete PV coverage is infeasible with post-construction installations, but not with newer technologies whereby the roof is an integrated solar panel^19^,^20^,^21^. Urban land use intensity is derived from satellite and population density based data classified into high (57% roof space hence solar panels, 38% roads, 5% vegetation), medium (45% solar panels, 45% roads, 10% vegetation), and low (25% solar panels, 25% roads, 50% vegetation) intensity development^22^ (Supplementary Figure S1). We report results for four different levels of solar panel efficiency: 20% (SD20) (now available with some recent commercial PV modules), 30% (SD30) (which is achievable with advanced module designs, 40% (SD40) (which has been achieved for concentrator PV systems^23^) and 60% (SD60) which represents a possible theoretical efficiency limit^24^).

**Figure 1:**
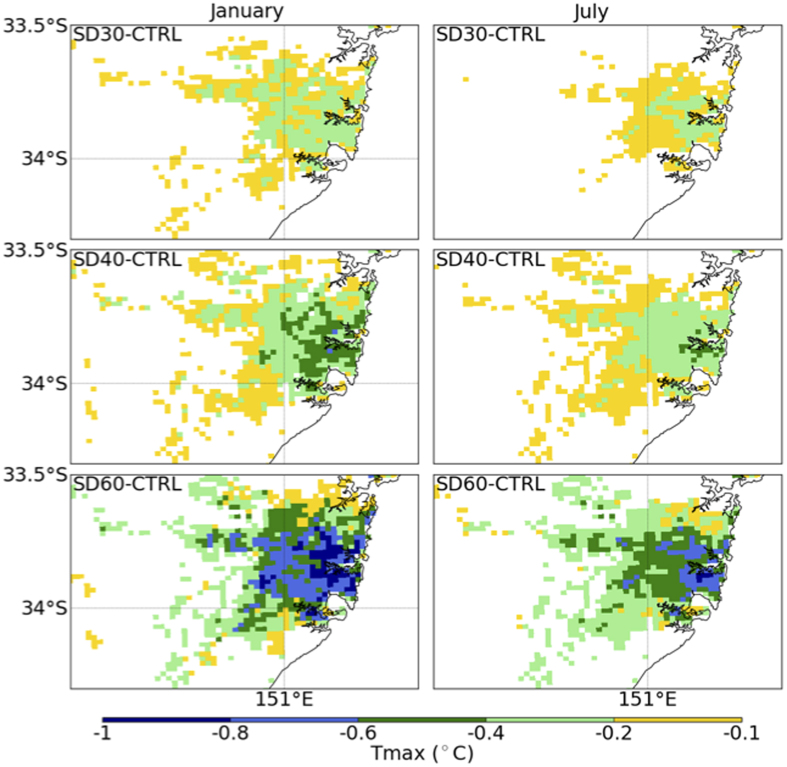
Impact on daily maximum temperature (°C) of installation of solar panels under the SD30 (top row), SD40 (middle row) and SD60 (bottom row) for January (left column) and July (right column). The results are shown as a difference from the control experiment where no solar panels were installed. Map was generated using Python Software Foundation. Python Language Reference, version 2.7.5 (Available at http://www.python.org).

We first focus on the physical impact of solar panels on January and July daily maximum temperature (*T*_MAX_) over Sydney, one of the world’s 100 largest urban areas. Cooling in SD30 occurring over the medium and high intensity urban areas is around 0.3 °C, reducing to 0.15 °C over the low intensity urban in January (Fig. 1, Supplementary Figure S2). In July, the cooling is less, but still reaches 0.3 °C. As the efficiency is increased, the impact on *T*_MAX_ strengthens in both January and July. For SD60, cooling approaches 1 °C in both January and July over high intensity urban surfaces, and 0.4–0.5 °C over low intensity urban surfaces (Fig. 1, Supplementary Figure S2). These reductions in *T*_MAX_ over Sydney are of the order of the warming observed to date^25^ due to anthropogenic emissions of CO_2_.

The changes in temperature are linked to changes in the surface energy balance. In January, the 151 W m^−2^ of net radiation (that is the net balance of solar and longwave radiation) is almost all used for two turbulent energy fluxes; principally the sensible heat flux (139 W m^−2^), and small amounts of latent heat fluxes (7 W m^−2^, Supplementary Figure S3). As solar installations increase, the net radiation increases due to the lower albedo, but sensible heating decreases as a consequence of the solar energy production (Supplementary Figure S3). The lower sensible heat flux reduces the heating of the lower atmosphere relative to the control simulation and consequently the atmosphere cools. Similar logic explains the July temperature changes (Supplementary Figure S3, second column).

These reductions in temperature are achieved via the generation of energy that offsets the need to import that amount of energy into the city. This leads to cooling since the energy used by the solar panels, which would have otherwise heated urban surfaces and then radiated energy into the atmosphere, is used to generate electricity that delivers residential, commercial and industrial energy services that would otherwise require the import of energy. This is a critical distinction with geo-engineering strategies such as painting surfaces white to reflect solar energy. We can derive an albedo that would cool via reflection of sunlight, but without the benefit of generating electricity that offsets the need to import energy. The temperature benefits accrued via SD20 could be matched by increasing the albedo of an equivalent area by ~0.08, increasing to ~0.17 for SD30, ~0.26 for SD40 and ~0.44 for SD60. As a guide, deforesting a tropical forest and replacing with a grassland increases albedo by ~0.1, providing a sense of how large an increase is required to match the impact of large installations of solar panels.

**Figure 2:**
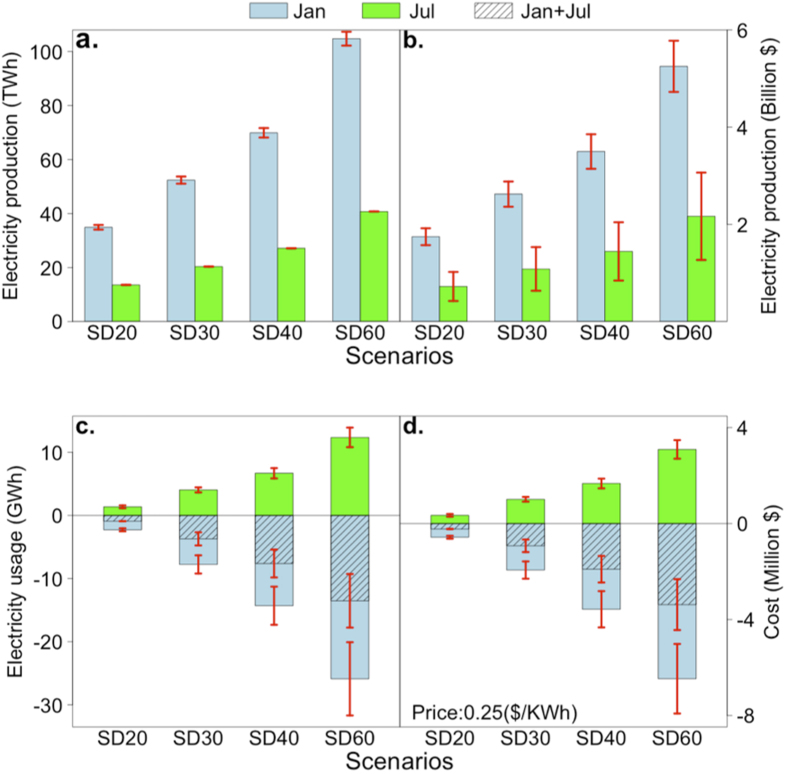
(**a**) Amount of energy produced (TWh, or 1 × 10^12^ Wh) for each solar panel installation scenario for January (blue), July (green) averaged over 2007 and 2009. The red bars show the range of the estimates for the individual years; (**b**) the estimated value of this energy at current prices; (**c**) estimated electricity saved (GWh, or 1 × 10^9^ Wh) for each solar panel installation for January (blue), July (green) and combined (hatched); (**d**) as previous panel but expressed as a value in AUD assuming current pricing of 25 cents per KWh.

There are two key financial implications of our result: a value associated with the energy generated and a value associated with the ambient cooling. Consistent with other studies^16^ energy produced is very large (Fig. 2a and Supplementary Figure S4) even under SD20 reaching 35 TWh in January and 13.5 TWh in July, suggesting an annual PV production from just the Sydney region of around 290TWh. By comparison, Australia’s annual electricity consumption is currently around 250TWh although total energy consumption is over six times this^26^ and one can envisage widespread substitution of electricity for other energy sources in a carbon constrained future^27^ or due to the widespread production of electricity from PV. An estimated total energy consumption (direct and indirect) of Sydney households is around 250TWh/year^5^. In terms of the financial value of this PV generation, current wholesale electricity prices in Australia average around $50/MWh (ref. 26) suggesting a value of PV generation under SD20 of around AUD$1.8b in January (Fig. 2b). PV generation increases to ~100 TWh under SD60 in January, suggesting annual Sydney PV production of around 750TWh, which is approaching half of current Australian energy consumption and highlights the stretch nature of these higher PV deployment scenarios. We do not explore the value of this electricity further given the profound market transformation that would result from such levels of PV deployment. We do price the value of the cooling impact of the solar panel installations to provide some sense of one potential financial saving associated with the reduced temperatures arising from major Sydney PV deployment.

Using historical electricity data from Australia’s largest and Sydney’s main distribution network service provider (Ausgrid) and temperature data from Sydney’s longest continuous meteorological station (Observatory Hill), we relate temperature with electricity use (see Methods). The estimated reduction in electricity usage due to the reduction in ambient temperature for January was 2–2.5 GWh under SD20 (the range relates to the differences between January 2007 and 2009), increasing to 6.3–9.2 GWh under SD30, 11.3–17.3 GWh under SD40 and 20.0–31.7 GWh under SD60 (Fig. 2c). In July, energy use increased by smaller amounts (Fig. 2d) such that the net impact was a saving in electricity of ~0.9 GWh under SD20, 3.7 GWh under SD30, 7.6 GWh under SD40 and 13.5 GWh under SD60 (Fig. 2c).

The change in electricity usage due to temperature changes was then priced using the current blended retail cost to businesses and homeowners of around AUD$0.25/KWh (ref. 26). The savings in January are between AUD$0.51 and $0.63 million for SD20, increasing to AUD$1.58-$2.3 million for SD30, AUD$2.8-$4.3 for SD40, and AUD$5.0-$7.9 million for SD60 (Fig. 2d). July costs increase, but the net value of the electricity ranges from AUD$0.23 million under SD20 to $3.38 million under SD60 (Fig. 2d). These savings relate to a reduction of ambient temperature, associated with cooling from solar panels, but the savings would be similar via an increased albedo of the magnitude noted earlier. However, unlike a change in albedo, the solar panels generate electricity, which is valuable commodity.

A warming city is a threat to its population. Heat waves and extreme temperatures lead to high mortality^28^,^29^ and increases in hospital admissions^30^,^31^. Increasing temperature extremes also threaten infrastructure; railway lines buckled and energy transmission failed in Victoria, Australia during a heat wave in January 2009 at an estimated cost of AUD$800 million^32^. Direct impacts of heat on human productivity have also been identified^33^. Cooling cities would therefore provide a variety of additional benefits, which are difficult to value but are potentially very large.

There are many possible strategies to cooling cities^7^ and we have shown that a large-scale installation of solar panels is effectively a localized geo-engineering strategy with particular advantages over other possible UHI reduction approaches. It can be targeted at populations at risk and generate offsetting financial benefits. In addition, maximum temperatures cool areas beyond the immediate vicinity of the panels even to where the panels are not (generating positive externalities), would reduce the risk of extreme temperatures to populations and infrastructure, and also provide further direct economic value. However, the impact on maximum temperature of even extreme solar panel deployment is less than 1 °C. While this would help, and would generate considerable energy that could be used for a range of purposes, the quantum of cooling is limited compared to projected warming due to human emissions of greenhouse gases by 2050. While we can match the impact of solar panels with albedo management via white roofs, our results suggest that it would require extremely high levels of albedo management to cool a city by 1 °C and that this strategy lacks the associated benefits of electricity production.

## Methods

### WRF and its urban model

We use Weather Research Forecast (WRF 3.7.1) to simulate the January (summer) and July (winter) climate of Sydney. WRF is an atmospheric model coupled to the land surface. Changes in the atmosphere (e.g. clouds) therefore affect the amount of energy received at the surface, and changes in the surface energy balance (including changes in the partitioning of net radiation between sensible heat and latent heat) affects the atmospheric temperature and humidity. We use a configuration of WRF that has been extensively tested over Australia^34^,^35^. WRF simulations use triple-nesting with three domains at 50 km, 10 km and 2 km resolution and we focus on the 2 km simulations (Supplementary Figure S1). This configuration uses the WRF Single Moment 5-class microphysics scheme; the Rapid Radiative Transfer Model (RRTM) long-wave radiation scheme; the Dudhia shortwave radiation scheme; the Monin-Obukhov surface layer similarity; the Noah land-surface scheme; the Yonsei University boundary layer scheme and the Kain-Fritsch cumulus physics scheme, Thompson micro-physics scheme. No cumulus physics is used for the 2-km simulation because most convection can be explicitly resolved. The single-layer Urban Canopy Model (SLUCM^36^,^37^) is used to represent urban surfaces and includes urban geometry which is represented through infinitely long street canyons, with various urban surfaces (roof, walls, and roads) to introduce different sensible heat fluxes. The effects of shadowing, reflections and trapping of radiation in street canyons are considered. SLUCM coupled with WRF has been extensively validated^38^.

### Solar panels

Conventional Photovoltaic (PV) panels are rapidly developing^16^ and concentrated PV panels (CPV) have emerged that use multi-junctions to achieve an efficiency of more than 40%. These are projected to achieve 60% efficiency in the next few years^23^,^24^. We therefore examined the utility of four different levels of efficiency: 20%, 30% 40% and 60% (SD20, SD30, SD40 and SD60). We assume that the albedo of the horizontally-installed solar panel^16^ is 0.1, otherwise the albedo of the urban surface^16^,^34^ is 0.2. This is the default value in WRF and was used to overcome the lack of direct observations of albedo for Sydney.

We note that we assume that solar panels cover 100% of available roof area, and vary solar panel efficiency. While this may have recently become technically feasible (e.g. www.tesla.com/solar), our core rationale is to examine the potential ability of solar panels. We therefore examined four scenarios with different efficiencies (20%, 30%, 40% and 60%). Equivalently, these four scenarios could represent a constant solar panel efficiency with varying degrees of roof coverage. If we assume that the efficiency of the solar panels is 60% for all of experiments, the effective solar panel coverage changes from 33%, 50%, 67% and 100% respectively for the experiments with efficiency at 20%, 30%, 40% and 60% and provides equivalent results.

In order to calculate the amount of electricity generated by the solar panels, we explored the impact of different levels of efficiency in converting direct shortwave radiation into power. Taking 30% efficiency as example, the 30% of the remaining 90% direct shortwave radiation after reflection was absorbed by the panels and converted to electricity, and the other 70% of the remaining 90% direct shortwave radiation transmitted through the panels and absorbed by the underlying surface. Thus the effective solar panel efficiency is 27% (90%*30%) for SD30.

### Pricing the value of urban cooling on power consumption

Using electricity data from a key Australian energy supplier (Ausgrid) and temperature data from Sydney’s longest continuous meteorological station (Observatory Hill), we relate temperature with electricity use for January 2007, July 2007, January 2009, and July 2009. Increased electricity use is associated with temperature increases in the summer (January) due to additional cooling needs, but is associated with temperature decreases in the winter (July) due to the need for additional heating as temperature falls. For each WRF grid point (~2 km^2^) where solar panels were added, temperature was associated with the closest Ausgrid power substation, and the average change in temperature simulated by WRF for each 2 km^2^ was associated with a substation for each day. The days were then separated into working and non-working days and the average changes in temperature from WRF for each substation for that day were multiplied by estimates from regressions of observed daily maximum temperature on observed energy use to estimate the total change in electricity usage for each substation for each day under different simulated scenarios. This change was summed across each substation and across the month to obtain a total change for that month in electricity usage.

## Additional Information

**How to cite this article:** Ma, S. *et al*. Pricing the urban cooling benefits of solar panel deployment in Sydney, Australia. *Sci. Rep.* **7**, 43938; doi: 10.1038/srep43938 (2017).

**Publisher's note:** Springer Nature remains neutral with regard to jurisdictional claims in published maps and institutional affiliations.

## Supplementary Material

Supplementary Information
