# Supplementary material for: Pricing the urban cooling benefits of solar panel deployment in Sydney, Australia
Source: Sci Rep. 2017 Mar 6;7:43938. doi: 10.1038/srep43938 (PMC5338272; doi:10.1038/srep43938)
Supplement: Supplementary Information [file srep43938-s1.doc]

**Pricing the urban cooling benefits of solar panel deployment in Sydney, Australia**

S. Ma^1^, M. Goldstein^2^, A.J. Pitman^1^, N. Haghdadi^3^, I. MacGill^4^

**Supplementary Figures**


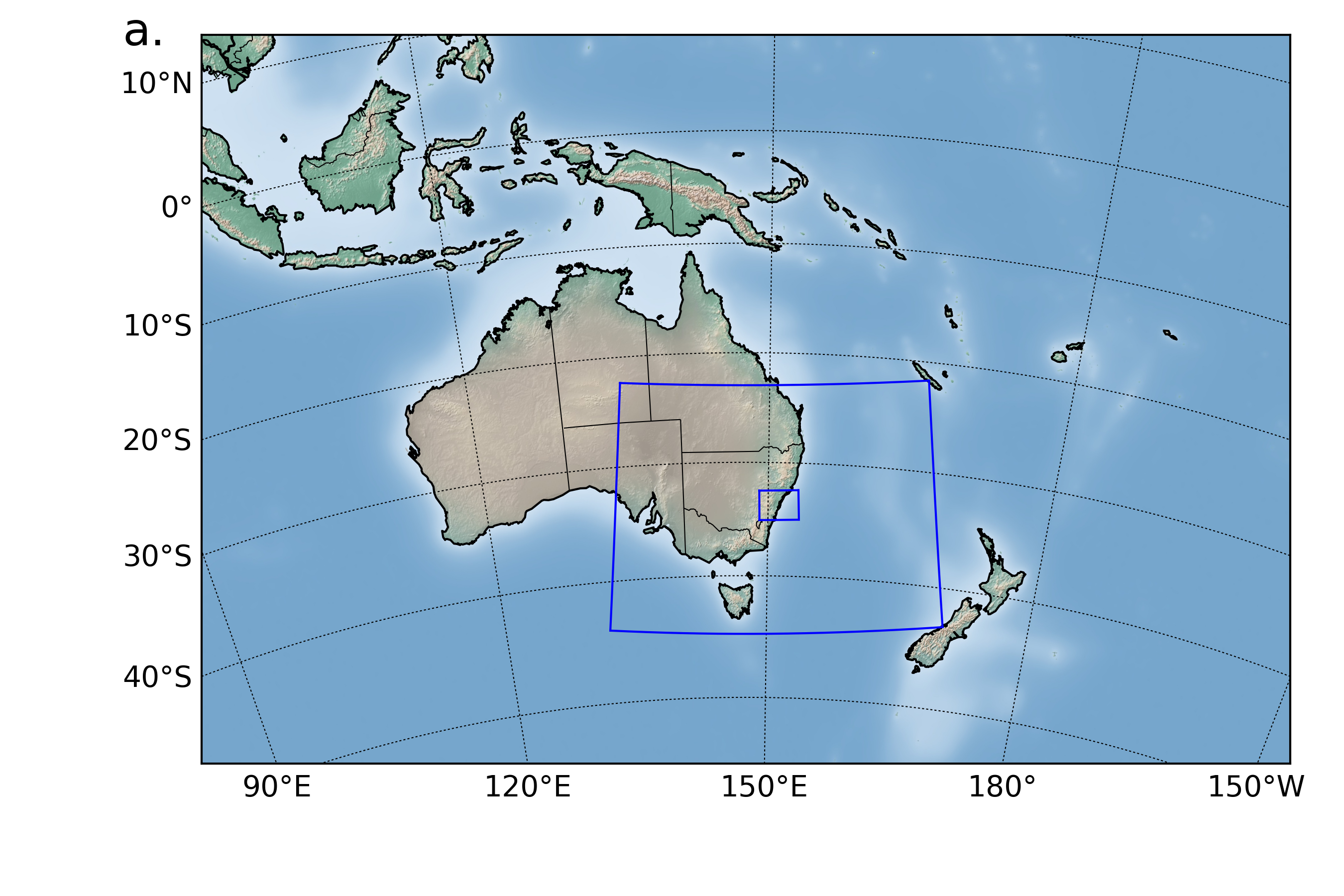


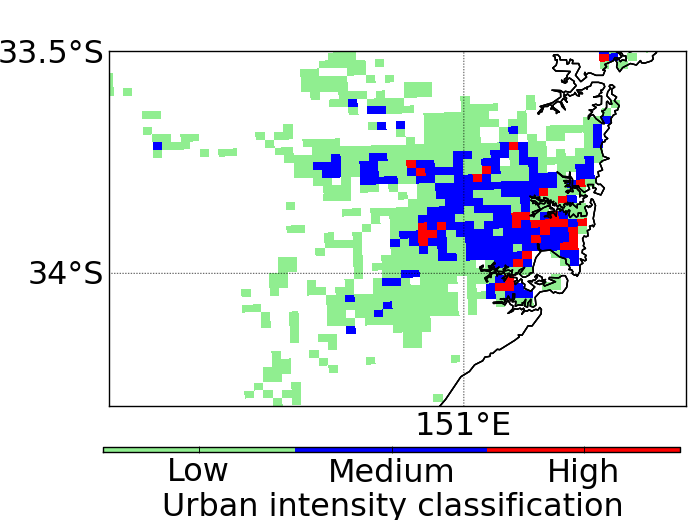


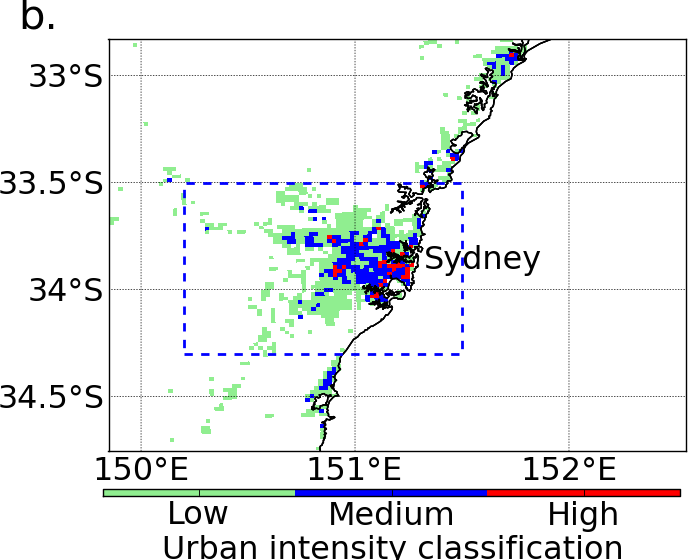


c.

*Figure S1 Details of the domains, and the density of the urban surface used by WRF. The large-scale domain is shown in (a) and is resolved at 50km. Two rectangles in (a) show the regions modeled at 10km (larger rectangle) and 2km (smaller rectangle). The smaller rectangle is shown in (b) which shown regions of urban land cover as low, medium and high density. The dashed rectangle shows the region used for diagnostic and illustrative purposes in other figures and using this region, (c) shows the distribution of low, medium and high density urbanization regions across Sydney. Map was generated using Python Software Foundation. Python Language Reference, version 2.7.5 (Available at http://www.python.org).*


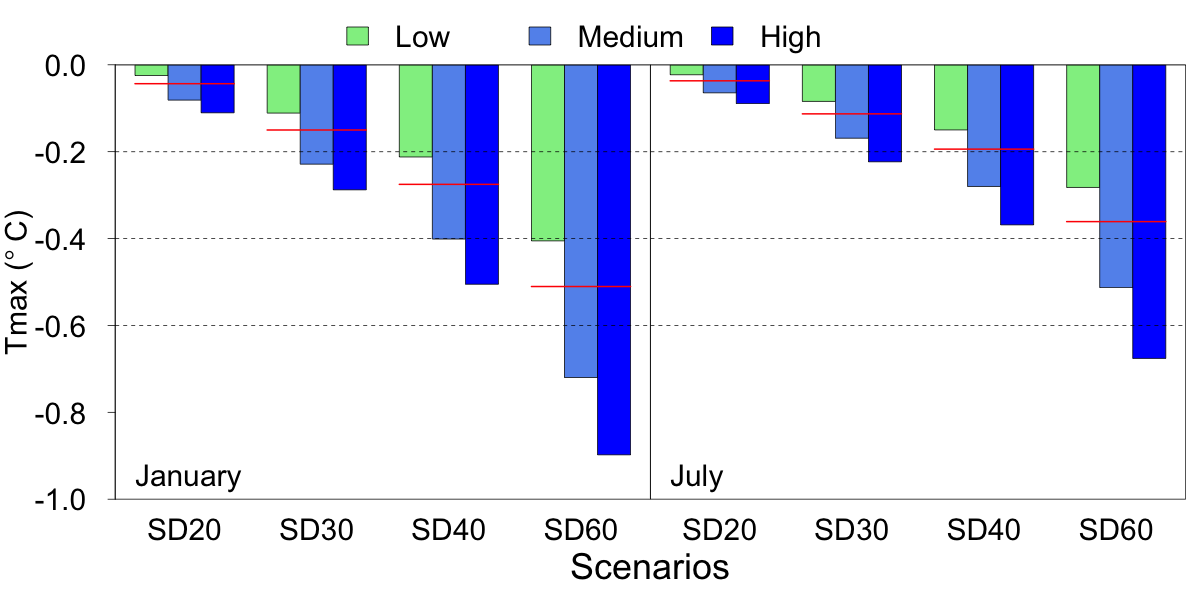


*Figure S2: Impact of solar panel installation on maximum temperatures across the high, medium and low urbanization regions shown in Supplementary Figure 1. Results for each experiment are shown for January (left) and July (right).*


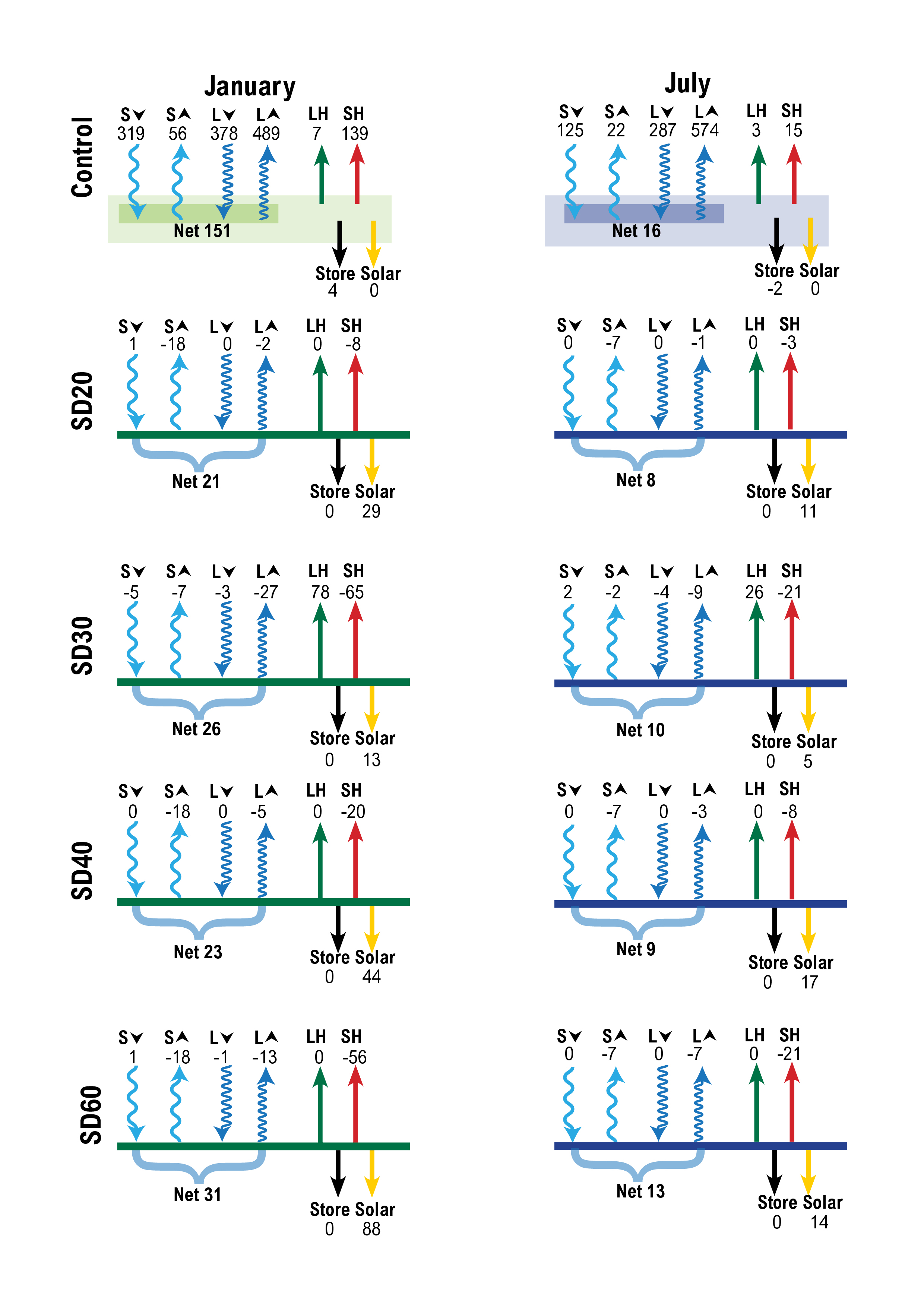


*Figure S3: Surface energy balance data for each experiment. The first row shows the data for the control experiment, subsequent rows show differences from the control for each experiment. The terminology is S (solar radiation), L (longwave radiation), Net (net radiation), LH (latent heat flux), SH (sensible heat flux), Store (storage) and Solar (energy generated via the solar panels). All data are in W m^-2^ and for S and L the downward and upward fluxes are identified by arrows. Figure was created using Adobe Illustrator CC (2017), Adobe Indesign CC (2017) and Adobe Acrobat DC.*

*
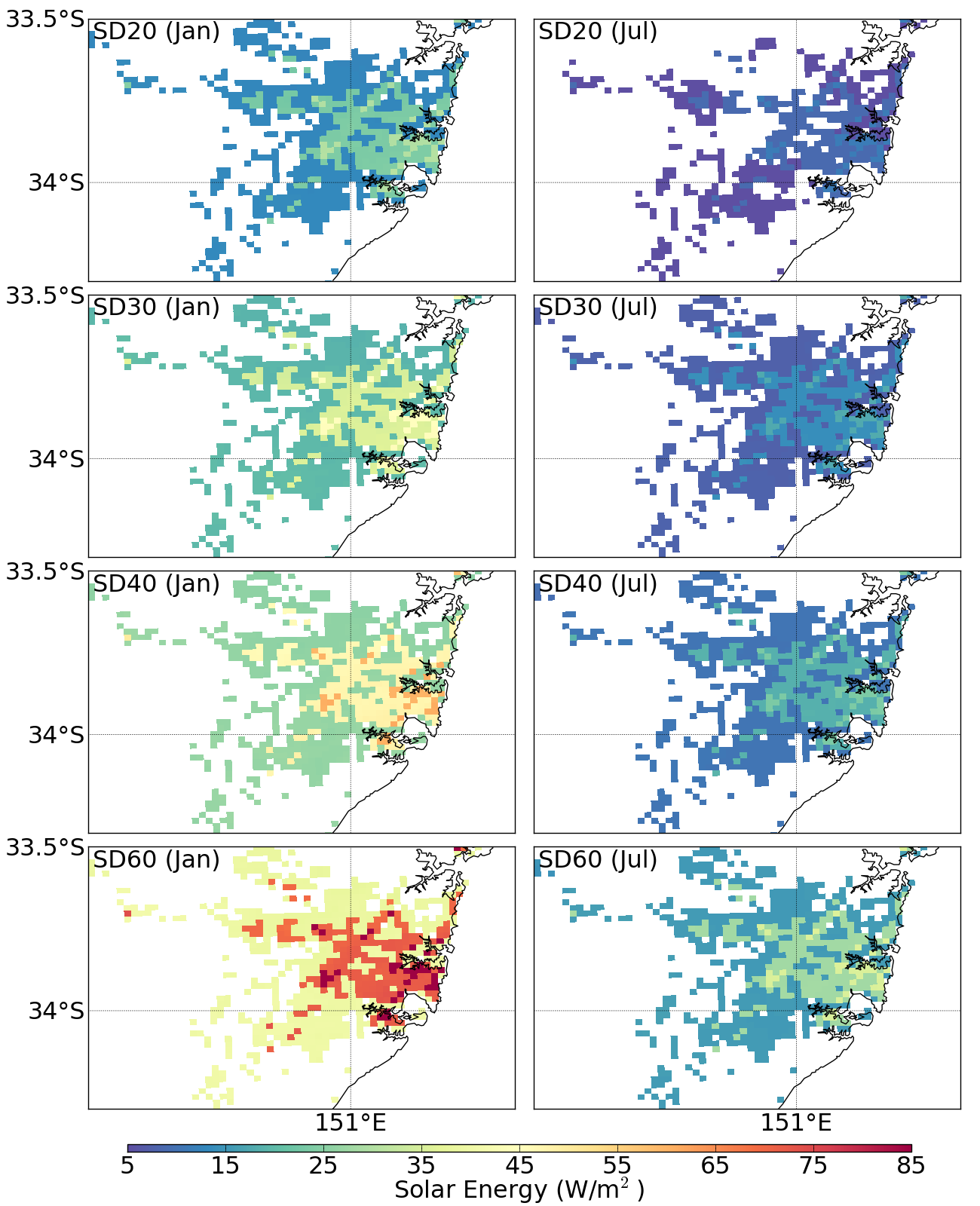
*

*Figure S4: Solar energy (W m^-2^) harvested in each experiment for January (left column) and July (right column). The patterns reflect the low, medium and high urbanization regions shown in Supplementary Figure 1. Map was generated using Python Software Foundation. Python Language Reference, version 2.7.5 (Available at http://www.python.org).*
